# Practical obstetric multi-professional training (PROMPT): the evidence for effective training

**DOI:** 10.1007/s00404-025-08213-1

**Published:** 2025-10-17

**Authors:** Spyridon Papageorgiou, Lars Brodowski, Halina Lewinski, Bettina Bohnhorst, Markus Flentje, Sven Schiermeier, Steven R. Talbot, Constantin von Kaisenberg

**Affiliations:** 1 Department of Obstetrics, Gynecology and Reproductive Medicine, 1-3 at the Hannover Medical School, Carl-Neuberg-Str. 1, 30625 Hannover, Germany; 2 Department of Pediatric Pneumology, Allergology and Neonatology, 1-3 at the Hannover Medical School, Carl-Neuberg-Str. 1, 30625 Hannover, Germany; 3 Department of Anaesthesiology and Intensive Care, 1-3 at the Hannover Medical School, Carl-Neuberg-Str. 1, 30625 Hannover, Germany; 4 Department of Obstetrics and Gynecology, Marien Hospital Witten, Marienplatz 2, 58452 Witten, Germany; 5 Institute for Laboratory Animal Science, Hannover Medical School, Carl-Neuberg-Str. 1, 30625 Hannover, Germany

**Keywords:** PROMPT training, Shoulder dystocia, Brachial plexus injury, Neonatal outcomes, Teamwork and communication, Maternal and neonatal safety, Simulation training

## Abstract

**Objective:**

To test the hypothesis that Practical Obstetric Multi-Professional Training (PROMPT) is effective training.

**Background:**

Multi-professional training in the labour ward has, in most cases, shown to be effective, in some cases not to be effective, and in some instances, it has worsened the outcome following the introduction of training. If training is to be performed, it should be adequate training. Thus, monitoring the outcomes is mandatory to determine if training is effective. Adjustments become possible to achieve improved outcomes.

PROMPT Training has 14 modules: Team working, Basic life support and maternal collapse, maternal cardiac arrest and advanced life support, maternal anaesthetic emergencies, foetal monitoring in labour, pre-eclampsia and eclampsia, maternal sepsis, major obstetric haemorrhage, shoulder dystocia, cord prolapse, vaginal breech birth, twin birth, acute uterine inversion, basic newborn resuscitation.

The concept involves scientifically written modules based on clinical studies, multi-professional training, central integration of teamwork and communication training and multi-professional training in the labour ward for obstetricians, midwives, neonatologists, anaesthetists and further professions involved.

**Design:**

Systematic literature review.

**Methodology:**

A systematic literature search of PubMed, Embase, Medline, Scopus, and the Cochrane Library was conducted for studies published between January 2000 and November 2024. Eligible studies evaluated PROMPT training and reported clinical outcomes, training effects, or cost-effectiveness. Forty-two studies met inclusion criteria, comprising randomised controlled trials, observational cohorts, and quasi-experimental designs. Methodological quality was assessed using the Cochrane Risk of Bias tool, and sensitivity analyses explored consistency across study types.

**Results:**

A total of 62 studies were identified, of which 42 met the inclusion criteria and were analysed across 14 PROMPT training modules; 20 publications were excluded. Of the eligible studies, 37/42 reported improvements in 8/14 modules, most notably in teamwork and communication, management of shoulder dystocia with reductions in brachial plexus injury, decreased rates of hypoxic-ischaemic encephalopathy and low 5-min Apgar scores, improved management of pre-eclampsia with increased magnesium sulfate use, reduced decision-to-delivery intervals for umbilical cord prolapse, and better outcomes in postpartum haemorrhage, breech and instrumental deliveries, maternal cardiac arrest, and neonatal resuscitation. Additional findings included reduced litigation costs and evidence of cost-effectiveness. Three studies demonstrated no significant improvement, one trial reported worsened 5-min Apgar scores after implementation in 12 Scottish maternity units, and one study showed mixed outcomes. At Hannover Medical School, our own data demonstrated substantially reduced adverse outcomes after 2½ years of PROMPT training.

**Conclusions:**

PROMPT Training effectively reduces adverse outcomes of rare but severe obstetric complications, if training is implemented using authentic PROMPT materials, team and communication training will be implemented, and training will be repeated annually in a multi-professional way.

## Introduction

Over the past decades, medical advances have resulted in low pregnancy and childbirth complication rates. However, rare but severe complications at birth lead to serious, long-lasting consequences for both the mother and her newborn. Many of these conditions have been considered fateful because the dogma has been that they cannot be trained.

The key to effective management of such conditions is the scientific basis for the training from clinical studies, teamwork, communication and a standardised approach using algorithms for all specialisations involved, using patient actors and simple props (mannequins) including midwives and obstetricians, paediatricians, and anaesthetists. This approach requires authentic training materials: course manual, trainer manual, algorithms, check lists, documentation forms, and lectures. However, the most critical key is training together as a team (‘those who work together should train together’), and training must be repeated at least annually in more than 90% of staff.

PROMPT was introduced in 2000 by a multi-professional team of obstetricians and midwives from the Department of Women's Health at Southmead Hospital in Bristol.

PROMPT Training includes 14 modules: Team working, Basic life support and maternal collapse, maternal cardiac arrest and advanced life support, maternal anaesthetic emergencies, foetal monitoring in labour, pre-eclampsia and eclampsia, maternal sepsis, major obstetric haemorrhage, shoulder dystocia, umbilical cord prolapse, vaginal breech birth, twin birth, acute uterine inversion, basic newborn resuscitation.

The effectiveness of the PROMPT concept has been published on the website of PROMPT New Zealand (https://www.promptnz.org/evidence-of-effectiveness), and there is now cumulative evidence of its effectiveness in various hospitals, countries and continents (for details, see also: http://www.promptmaternity.org, the global network).

This systematic review aims to test the hypothesis that PROMPT is effective training by analysing the published evidence in multi-professional training.

## Methodology

A systematic literature search was conducted from November 1st, 2024, to December 1st, 2024, using the following search terms: obstetric emergency training, obstetric simulation, teamwork, litigation costs, cost-effective training, knowledge, skills, and teamwork in association with PROMPT. PubMed, Embase, Medline, Scopus, and Cochrane databases were systematically searched for the period spanning January 1, 2000, to November 1, 2024.

**Fig. 1:**
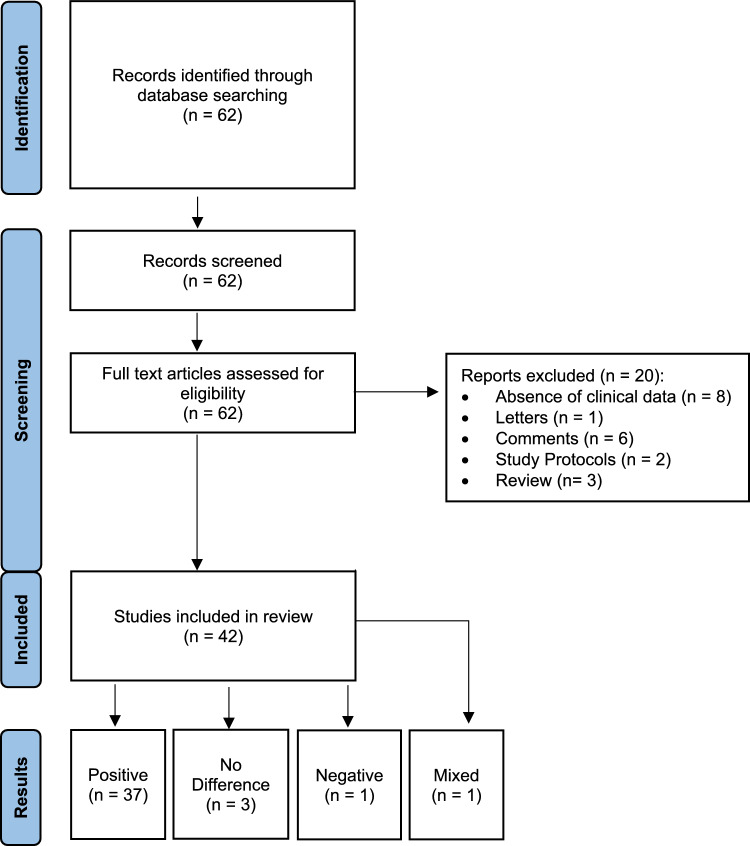
Literature search results

**Fig. 2:**
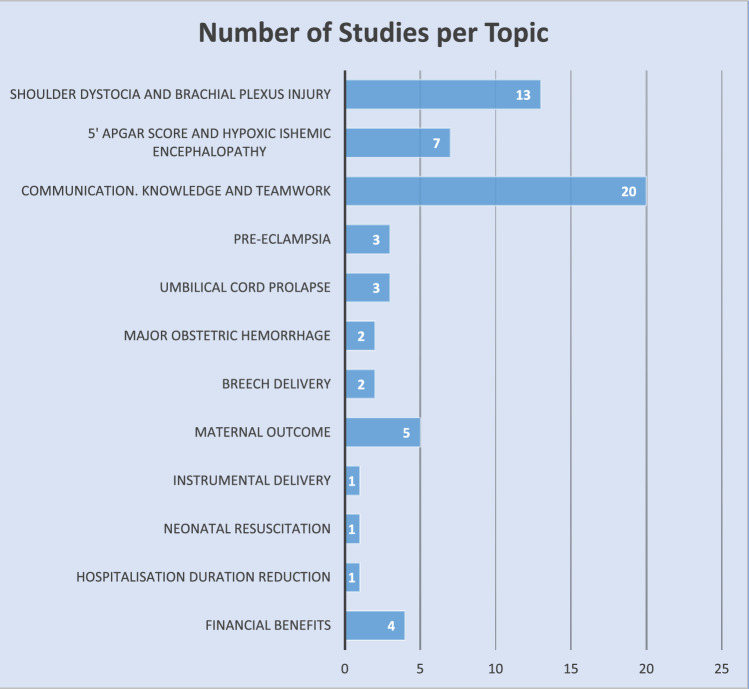
Number of citations according to topic

The resulting citations were reviewed by title, abstract, and full text, as necessary, through three investigators (SP, CvK, SS). The process spanned two weeks, from December 1st to December 15th, 2024. Citations were excluded based on non-obstetric evidence, the absence of training results, and/or the absence of financial data. The resulting articles were then reviewed for inclusion using the criterion implementation of PROMPT Training with an emphasis on the clinical outcomes, the trainings’ protocol, the impact on litigation costs, and the cost-effectiveness. (Table 1) Randomised control trials, observational and cohort studies, and quasi-experimental studies were prioritised in the literature review. Review articles that contributed novel insights to the field were also included. The inclusion process is depicted in the Prisma flowchart below (Fig. 1) and organised into groups based on the topic of each study (Fig. 2).

**Table 1:** Inclusion / Exclusion Criteria

Inclusion criteria	• multi-professional training (PROMPT) • clinical outcomes • training protocols • impact on litigation costs • cost-effectiveness (training vs litigation costs) • RCTs • observational & cohort studies • quasi experimental studies
Exclusion criteria	• non-obstetric evidence • absence of training results, clinical & financial data • letters & comments • study protocols

### Risk of bias and sensitivity analysis

We assessed the quality of the included studies using the Cochrane Risk of Bias tool. This tool looks at several aspects of study design, such as how participants were randomly assigned, whether group allocation was concealed, whether participants and assessors were blinded, whether outcome data were complete, and whether all planned outcomes were reported. Each of these areas was rated as having a low, high, or unclear risk of bias. To check how reliable our results were, we also carried out a sensitivity analysis. This involved grouping studies by design (randomised trials, interrupted time-series, cohort, cross-sectional, or simulation-only) and re-analysing the results after removing studies judged to be at high risk of bias. This helped us see whether the overall conclusions were stable across different study types or overly affected by weaker evidence.

## Results

### Effective training/improved outcomes

Ten UK studies assessed the impact of PROMPT training on neonatal outcomes before and after implementation. PROMPT was effective in simulation, improving successful shoulder dystocia deliveries from 50 to 85% [1] and 42.9% to 83.3% [2], with appropriate manoeuvre use increasing from 46.3% to 99.8% [3].

Three studies (Crofts et al. [3]; Draycott et al. [4]; Weiner et al. [5]) tested whether PROMPT reduced brachial plexus injury (BPI) during shoulder dystocia. All found significant reductions (*p* < 0.05) in BPI percentage and relative risk (Table 2), with Weiner et al. reporting no permanent injuries. Bristol has maintained a zero permanent BPI rate for 10 years.

**Table 2:** Brachial plexus injury in shoulder dystocia pre- and post-PROMPT training

	Shoulder dystocia incidents	Brachial plexus injury (vaginal)	Relative risk reduction of BPI %	*p*-value
1996–1999 (pre)	324 (15,908) 2.04%	39 (324) 12.04%		
2001–2004 (post)	262 (13,117) 2.00%	10 (262) 3.82%	68.1%	0.04
2008–2010 (pre)	113 (3285) 3.44%	10 (113) 8,5%		
2011–2012 (post)	95 (2589) 3.67%	0 (95) 0%	100%	< 0.01
2009–2012 (post)	562 (17,073) 3.29%	8 (562) 1,42%		
Total (pre)	437 (19,193) 2.28%	49 (437) 11.21%		
Total (post)	919 (32,779) 2.80%	18 (919) 1.96%	82.5%	0.009

Furthermore, under clinical conditions, BPI fell from 9.3% to 2.3% [4, 6] and rates of low Apgar scores and hypoxic-ischaemic encephalopathy (HIE) were reduced by about 50% [7] (Table 3), improving overall shoulder dystocia management [1–4, 7–10]. Theoretical knowledge also increased by 92.5%, reflected in higher MCQ test scores after training [10, 11].

**Table 3:** Improvements in neonatal outcome pre and post-PROMPT Training

Neonatal outcome	1998–1999 (pre)	2001–2003 (post)	Relative risk reduction	*p*-value
5´ APGAR-score < 6	73 (8,430) 0.86%	49 (11,030) 0.44%	51%	< 0.001
Hypoxic ischaemic encephalopathy (HIE)	23 (8,430) 0.27%	15 (11,030) 0.14%	50%	0.03
Moderate/severe HIE	16 (8,430) 0.19%	11 (11,030) 0.10%	53%	0.09

A UK RCT comparing eclampsia training in local hospitals and a regional simulation centre found significant gains in all aspects of care, with basic task completion rising from 87 to 100%, magnesium sulphate use from 61 to 92% [12], and administration occurring 2.5 min faster [13]

Simulation for postpartum haemorrhage (PPH) showed mixed findings: Deering et al. [14, 15] reported only 45% corrected haemorrhage within 5 min and 47.5% made medication errors, whereas Maslovitz et al. and Siassakos et al. demonstrated improved management, sustained at 6 months [16, 17].

For cord prolapse, PROMPT training reduced the diagnosis-to-delivery interval from 25 to 14 min, lowered low Apgar rates from 6 to 0%, and decreased NICU admissions from 38.5% to 22.2% [18] (Table 4). Croft et al. [2, 10, 12, 19, 20] also showed significant knowledge improvements pre- and post-training (Table 5).

**Table 4:** Improved umbilical cord prolapse management pre and post-PROMPT

Umbilical cord prolapses	1993–1999 (pre)	2001–2007 (post)	*p*-value
Number of eligible cases	*34*	*28*	
Diagnosis-delivery time	25‘	14‘	< 0.001
Actions to alleviate cord compression	34.78%	82.35%	0.003
Low APGAR-score (5´ < 7)	6.45%	0%	0.302
Admission to NICU	38.46%	22.22%	0.210

**Table 5:** The effect of PROMPT on the knowledge and skills of the trainees

Knowledge level of trainees	Before training	After training	Relative risk (difference in ability)	*p*-value
Participants score in multiple choice test (*N* = 133) [10]	80.4 (185) 43.5%	101.0 (185) 54.6%	20.6%	0.03
Shoulder dystocia simulation trainees achieving delivery [2]	60 (140) 42.9%	110 (132) 83.3%	48.5%	< 0.001
Teams of participants completing basic tasks^1^ [12]	20 (23) 87%	24 (24) 100%	13%	NP^2^
Teams preparing loading dose of Magnesium (LDM) [12]	17 (23) 74%	23(24) 96%	23%	0.06
Teams administering LDM [12]	14 (23) 61%	22 (24) 92%	33.7%	0.04
Teams preparing maintenance DM [12]	4 (23) 17%	9 (24) 38%	55.3%	0.06

^1^Basic task: called for help, stated problem, called anaesthetist, lowered headrest, recovery position, oxygen administrated

^2^NP: It is impossible to test because there were no disjoint pairs or only one

In the USA, a 5-year Kansas analysis showed a 6% drop in C-section rates, better recognition of shoulder dystocia, and BPI reduction from 10.7% to 0% [5]. Smith’s Baltimore study reported shorter delivery times (146 s to 61 s), more frequent calls for help (35% to 94%), paediatrician attendance (8% to 75%), and improved patient communication (57% to 83%) [21].

Internationally, PROMPT was introduced in 8 Australian units in 2008 [22]. Shoushtarian et al. [23] studied 43,408 births, reporting significant reductions in 1’ Apgar < 7 (9.1% to 7.7%), cord lactate > 5.27 mmol/l, and mean hospital stay (2.85 to 2.79 days), though 5’ Apgar < 7 and major PPH remained unchanged. PROMPT implementation in New Zealand (2016) is ongoing, while in South Africa, early structured teaching without practical training increased morbidity and mortality. In particular, deaths due to obstetric haemorrhage had increased by 40%, and 80% of the haemorrhage deaths were assessed as possibly or probably avoidable. However, later adoption of ESMOE (based on PROMPT) reduced maternal deaths by 11% between 2011–2013 [24].

PROMPT also improved teamwork and safety culture. Retrospective analyses reported higher ward safety culture scores (*p* = 0.036), improved teamwork climate and job satisfaction (*p* = 0.052), and significant gains in doctors and nurses “working well together” (*p* < 0.001) [25]. Qualitative work identified leadership, communication, and patient-centred care as essential [26] [27] and defined key leadership practices: recognising the situation, allocating tasks with closed-loop communication, and maintaining patient focus [28].

The training model applied also influenced outcomes. Studies comparing patient–actors vs mannequins found significant improvements in all scores (*p* = 0.017–0.001), with patient–actor groups reporting higher safety (*p* = 0.048) and communication (*p* = 0.035) ratings [29]. High-fidelity mannequins [30] also showed advantages: higher delivery success (42.9% to 83.3%), reduced applied force, and better patient communication [31] (Table 6). Despite higher cost, both methods improved outcomes, and training sustainability was emphasised [32, 33].

**Table 6:** Different results between using high- and low-fidelity mannequins

	High-fidelity mannequin (post-training) N^1^ (64)	Low-fidelity mannequin (post-training) N^2^ (68)	*p*-value
Successful delivery rate	94%	72%	0.002
Applied force	2,030 Newton seconds	2,916 Newton seconds	0.006
Call for paediatric support	22%	47%	0.003

A critical issue for healthcare organisations is the cost of implementing and maintaining multi-professional obstetric emergency training. Several retrospective and economic studies have evaluated the expenses associated with PROMPT and its impact on reducing litigation and adverse outcome costs.

In the United States, a seven-year analysis demonstrated substantial reductions in compensation payments after PROMPT introduction. Average annual perinatal claims fell from US$27.6 million (2003–2006) to US$2.6 million (2007–2009). Adjusted to 2024 values, this decline represents a reduction from ~ US$43.7 million to ~ US$3.7 million annually, a saving of ~ US$40 million per year. A Kansas evaluation showed avoided costs were mainly from fewer cases of hypoxic-ischaemic encephalopathy (HIE) and reduced caesarean sections: four HIE cases prevented, valued at US$26.8 million (2003 USD; ~ US$44.4 million in 2024 values), and 645 fewer caesareans, valued at US$4.5 million (2011 USD; ~ US$6 million in 2024 values). Reductions in brachial plexus injuries were not included due to uncertainty in case valuation [21, 34].

Similar benefits were observed in the United Kingdom. PROMPT implementation was linked to a ~ 91% reduction in obstetric litigation costs [35]. At Southmead Hospital in Bristol (≈6,500 births annually), annual programme costs were €148,806 (~ €22,000 per 1,000 births), of which ~ 89% represented staff release time. Despite this, reductions in BPI and HIE generated annual savings of €7.5 million and €25 million, respectively, corresponding to a total saving of €32.5 million—a > 200-fold return on investment.

In Australia, PROMPT implementation supported by the Victorian Managed Insurance Authority was estimated to reduce litigation costs by more than 20 times the programme’s cost[23], again suggesting both economic and clinical benefits.

By contrast, the Dutch TOSTI cluster RCT modelled cost-effectiveness rather than relying on litigation data [36]. It compared four strategies: no training, centre-based training alone, training plus one onsite refresher, and training plus three refreshers at three, six, and nine months. The model found refresher frequency critical: one onsite refresher prevented ~ 8 adverse outcomes per 1,000 deliveries (ICER €3,432), while three refreshers prevented ~ 11 (ICER €5,115). For shoulder dystocia trauma, three refreshers reduced ~ 2 cases per 1,000 (OR 0.50; 95% CI 0.25–0.99), at an ICER of €22,878, with training costs of €25,546 for the initial course and €9,035 for each refresher.

Overall, despite upfront costs, PROMPT has consistently proven cost-effective. In systems with litigation data (US, UK, Australia), savings greatly exceeded expenses, while modelling from the Netherlands emphasises the additional value of regular refresher training.

PROMPT training was implemented at the Hannover Medical School (Germany) in 2017. Our 16-year analysis [37] from 2004 to 2020 has shown a reduction of BPIs associated with shoulder dystocia (from 14.6% to 4.3%) with zero permanent BPIs after the training and an increased initiation of manoeuvres. Furthermore, we observed a reduction of episiotomies and an increase of perineal tears III°/IV° in cases with shoulder dystocia. (Tables 7, 8).

**Table 7:** Shoulder dystocia cases and manoeuvres applied (mothers)

	Group		*p*-value
	before training	After training	
Shoulder dystocia total cases	48/18031 (0.27%)	23/4609 (0.50%)	0.017
McRoberts’ manoeuvre	37/48 (77.1%)	23/23 (100%)	0.013
Suprapubic pressure	13/48 (27.1%)	7/23 (30.4%)	0.784
Internal rotation & manual arm extraction	6/48 (12.5%)	5/23 (21.7%)	0.319

**Table 8:** Brachial plexus injury, asphyxia, and adverse outcomes (infants)

	Group		*p*-value
	before training	After training	
Total brachial plexus injury (BPI)	7/48 (14.6%)	1/23 (4.3%)	0.261
Permanent BPI	1/7 (14.2%)	0/1 (0%)	n.a. ^∗^
Total cases complicated with asphyxia	3/48 (6.3%)	4/23 (17.4%)	0.23
Adverse outcome after one year	0/3 (0%)	0/4 (0%)	n.a. ^∗^

n.a.*: The statistical significance calculation is unavailable for these Groups (0 cases in Group After Training)

### No outcome improvements

Several studies reported that obstetric emergency training did not translate into measurable improvements in patient outcomes, despite sometimes enhancing simulated performance.

In a multisite cross-sectional study based on the SaFE programme [38], 114 clinicians in 19 six-person teams managed a simulated eclampsia case while blinded assessors coded team behaviours. Teams that administered magnesium sulphate within 10 min—the efficiency benchmark—were characterised by earlier verbal declaration of the emergency (Kendall’s tau_b_ – 0.53, 95% CI – 0.74 to – 0.32; *P* = 0.004) and more frequent closed-loop communication during the critical task (tau_b_ 0.46, 95% CI 0.17–0.74; *P* = 0.022). They also had fewer room exits (median 3, IQR 2–5) compared with less efficient teams (median 6, IQR 5–6; *P* = 0.03). These findings highlight specific behaviours that improve simulated performance but, crucially, did not provide evidence of improved clinical outcomes in real patients.

A related analysis of the SaFE trial [39] explored whether team efficiency correlated with individual knowledge, skills, or attitudes. Among the same 19 complete teams (114 participants), the Magnesium Administration Rank (MAR)—a validated efficiency ranking tool—showed no association with team MCQ knowledge scores (including eclampsia-specific items, all Kendall’s tau_b_ values < 0.3 and non-significant), manual skills assessments, or safety/attitude measures across six domains. This indicates that higher individual competence or safety awareness did not predict whether a team would function efficiently under simulated pressure.

Broader evidence also suggests limited impact of interactive training on patient outcomes. The Cochrane Review by Merriel et al. [40] synthesised 11 randomised or cluster-randomised trials, covering approximately 2,000 healthcare providers and over 300,000 patients across obstetric, neonatal, trauma, and resuscitation emergencies. Owing to marked heterogeneity, the authors could not conduct a pooled meta-analysis. Instead, structured narrative synthesis indicated that interactive training may make little or no difference to survival (low-certainty evidence), and effects on morbidity, adherence to protocols, clinical practice, and organisation of care were very uncertain (very low-certainty evidence). Importantly, prespecified subgroup analyses did not identify consistent moderators of effectiveness, such as training setting (simulation centre vs local), multidisciplinary participation, proportion of staff trained, or leadership style.

Taken together, these findings underscore that while obstetric emergency training can foster better teamwork behaviours and improved confidence, this does not necessarily translate into improved maternal or neonatal outcomes. Evidence from both simulation-based studies and large systematic reviews highlights a persistent gap between enhanced provider performance in training environments and measurable changes in patient-level outcomes in clinical practice.

### Worsened outcomes

#### THISTLE stepped-wedge cluster RCT (Scotland)

The THISTLE trial [41] evaluated the health-service implementation of PROMPT on the proportion of term infants with Apgar < 7 at 5 min across 12 randomised maternity units (plus a supplementary analysis across all 15 large units). Among 87,204 eligible births (99.2% with Apgar recorded), 1,291 infants had Apgar < 7 at 5 min (overall 1.49%), with crude rates rising from 1.32% pre-intervention to 1.59% post-intervention. In the intention-to-treat (ITT) model adjusted for time, there was a non-significant reduction (OR 0.79, 95% CI 0.63–1.01). Because some units started earlier than their allocated step, others delayed, and two did not implement PROMPT at all, the authors also ran an as-implemented analysis using the actual local start date; this showed no evidence of improvement (OR 1.01, 95% CI 0.84–1.22). Extending the analysis to all 15 large Scottish units likewise found no intervention effect. Units with historically high Apgar < 7 rates remained higher during the study (OR 2.09, 95% CI 1.28–3.41). Duration of exposure (≤ 6, 6–12, 12–18, 18–24, ≥ 24 months) did not alter outcomes (overall LR χ^2^ = 3.7, *p* = 0.86).

#### Implementation and protocol differences reported by the trial

Local implementation varied substantially: some units omitted core PROMPT elements (e.g., foetal monitoring sessions; in several programmes shoulder dystocia training was also absent), and the content and frequency of courses differed between sites. The investigators could not measure the proportion of staff trained by unit (baseline staffing denominators were unavailable), which limited assessment of “intervention penetration”. The team noted that it was unlikely Scottish units trained 90–95% of staff within 12 months (coverage levels previously associated with favourable outcomes in other settings). They also described logistical constraints (limited implementation support; departures from the randomisation schedule; competing national initiatives, including a separate foetal monitoring programme) as part of the observed heterogeneity. The authors concluded that scaling up PROMPT across multiple Scottish maternity units was more challenging than expected, and therefore their results reflect real-world effectiveness under variable implementation rather than the full efficacy that might be achieved with complete and authentic delivery of the programme.

#### TOSTI (Netherlands): effectiveness signals and increased interventions

The TOSTI programme [36] compared four strategies: no training; a single simulation centre course; simulation centre course + one onsite refresher at 6 months; and simulation centre course + three onsite refreshers (3, 6, 9 months). In the embedded trial/economic model, the primary analysis showed increased use of > 4 units blood transfusion and higher rates of embolisation or hysterectomy for postpartum haemorrhage in the intervention group. The authors state these PPH treatment increases were encouraged by the training and likely represent behaviour change (Kirkpatrick level-3) rather than a deterioration in patient outcome, and therefore excluded “severe PPH” from the composite patient-outcome measure used for cost-effectiveness. In contrast, neonatal trauma due to shoulder dystocia was halved (OR 0.50, 95% CI 0.25–0.99). In modelling, the benefits of training diminished after approximately three months, making repetition essential. More frequent refresher sessions not only reduced adverse outcomes but also remained cost-effective, as noted above.

Across Scotland, PROMPT as locally implemented did not reduce Apgar < 7 at 5 min; heterogeneity in timing, content, fidelity, and staff coverage was documented, and exposure duration was not associated with improvement. In the Netherlands, simulation-based team training showed mixed signals: more active PPH interventions following training (interpreted as intended practice change) alongside a reduction in shoulder dystocia–related neonatal trauma, with modelling indicating benefit only when refreshers were delivered at regular intervals.

## Discussion

### Long-term impact and sustainability of PROMPT training

Evidence indicates that a single PROMPT training session is insufficient for long-term skill retention. Crofts et al. [3] showed that while many participants maintained skills at 6–12 months, others required more frequent rehearsal, and reported measurable knowledge decay within six months. [11] Sustained benefits have been documented in long-term programmes, including a 12-year reduction in brachial plexus injuries in the UK [3] and multi-year improvements in US hospitals [5, 34]. The Dutch TOSTI trial [36] further demonstrated that multiple refreshers were more effective than a single course, preventing up to 11 adverse outcomes per 1,000 births. While these findings highlight PROMPT’s effectiveness as a recurrent, institutionalised programme, frequent refreshers may be resource-intensive and difficult to sustain in lower-resource settings [23, 33, 42].

### Barriers to adoption in low-resource settings

Despite its demonstrated benefits, PROMPT faces barriers to widespread adoption, especially in low-resource settings. Sustainability is challenged by the high cost of staff release and resource demands [33, 35] with even well-funded systems reporting incomplete staff coverage [23] and variable fidelity [41] In South Africa, Moran et al. [24] described major logistical and financial obstacles during the rollout of ESMOE, a PROMPT-based programme, while Ghag et al. [43] documented adaptations in the Philippines and cited evidence from Zimbabwe showing feasibility when PROMPT is locally tailored. These findings suggest that PROMPT can be suitable for low-resource contexts, but long-term success requires simplified delivery, local faculty development, and strong institutional support.

### Training equipment and fidelity

The question of whether high-fidelity mannequins are essential has been addressed in several studies. Crofts et al. [31] showed that both high- and low-fidelity simulators improved shoulder dystocia management, with only minor additional benefits from high-fidelity, while Ellis et al. [44] concluded that lower-cost, in-house solutions are often sufficient. Patient–actor-based training has also been shown to enhance communication and safety perceptions [45, 46] and cost analyses demonstrated that simple props such as “magic trousers” can be highly effective at a fraction of the cost of advanced simulators [35]. Taken together, these findings suggest that high-fidelity mannequins are not essential for successful PROMPT implementation, and that lower-cost alternatives may be more sustainable, particularly in resource-limited settings.

### Healthcare infrastructure and training outcomes (NHS vs Germany)

The impact of PROMPT depends in part on system infrastructure. In the NHS, multiple independent reviews have stressed that protected time and backfill are prerequisites for effective multidisciplinary training; the Ockenden Review explicitly calls for “sufficient protected time… including routine refresher courses,” a point echoed by the Royal College of Obstetricians and Gynaecologists and parliamentary reports highlighting the need for ring-fenced education funding and staff cover [47–49]. Even so, achieving high staff coverage remains challenging in practice (e.g., ~ 50% trained in a statewide rollout despite insurer support), and variable local fidelity can blunt impact [23, 41]. Within the NHS, unit configuration also shapes training needs: many Trusts run both alongside (AMU) and freestanding midwifery-led units (FMU), as well as home-birth pathways, which increases the logistics of coordinating joint drills and may necessitate more frequent sessions (e.g., monthly in high-volume centres) to reach rotating staff across sites [50, 51]. Cost analyses from PROMPT programmes show the opportunity cost is dominated by staff release time (≈90%), underscoring why backfill funding and leadership endorsement are often the decisive enablers [35].

In Germany, births are highly hospital-based with very low out-of-hospital rates (~ 2%), and the system comprises many smaller obstetric units compared with England; ongoing hospital consolidation and DRG-linked economic pressures have reduced the number of departments and exacerbated staffing constraints, which can make ring-fenced training time difficult and threaten programme continuity [52–54]. These structural differences imply that large NHS units (often several thousand births per year) may require higher-frequency drills to maintain coverage across large teams and multiple sites, whereas German units, being smaller and more numerous, may favour locally adapted, low-cost delivery (patient–actors, simple props, “course-in-a-box”) to minimise travel and release time while sustaining refreshers—approaches supported by PROMPT costings and implementation papers [32, 35]. Practically, therefore, infrastructure dictates the playbook: NHS trusts benefit from centrally mandated protected training time and robust attendance targets, but must tackle scale and rotation across AMUs/FMUs; German providers may achieve better uptake with decentralised, low-fidelity formats and shorter, more frequent sessions integrated into rosters, reducing the income/time penalty for clinicians while preserving the multidisciplinary component that underpins PROMPT’s effectiveness [29, 55].

### Risk of bias

The overall quality of the included studies was moderate to high, though several methodological limitations were noted. Most randomised controlled trials [29, 31, 36] demonstrated appropriate randomisation procedures but were limited by difficulties in blinding participants and outcome assessors, an inherent challenge in simulation-based interventions. Several observational designs, including large interrupted time-series and retrospective cohort analyses [4, 23, 34], provided robust longitudinal data but were susceptible to secular trends and potential confounding factors such as parallel safety initiatives. Smaller single centre studies often relied on surrogate outcomes (knowledge scores, simulated performance) rather than clinical endpoints, raising concerns about indirectness. Reporting bias was also a concern, as some studies lacked detailed statistical reporting of negative or neutral findings [42, 43].

### Sensitivity analysis

Sensitivity analysis revealed important differences between study types. Randomised controlled trials were most consistent in showing improvements in process outcomes, such as increased magnesium sulphate administration in eclampsia [39] or faster emergency response times [36], though effects on patient outcomes were variable. Notably, the large Scottish THISTLE RCT [41] found no improvement and even a worsening trend in 5-min Apgar scores, underscoring the influence of implementation fidelity. In contrast, interrupted time-series and cohort studies provided the strongest evidence of sustained patient benefit, with reductions in brachial plexus injuries and hypoxic-ischaemic encephalopathy observed across multi-year evaluations in the UK, US, and Australia [4, 23, 34]. Simulation-only and knowledge-retention studies consistently demonstrated improved technical performance and theoretical understanding [3, 8] but their translation into clinical outcomes was less certain. Taken together, these findings suggest that while controlled trials capture process gains, larger longitudinal and real-world studies demonstrate the most convincing improvements in patient outcomes, particularly when PROMPT is embedded as a recurrent institutional programme.

## Conclusion

PROMPT training has been shown to improve clinical performance and outcomes, particularly by reducing brachial plexus injuries, enhancing emergency response times, and strengthening teamwork. While most evidence is positive, some trials, such as THISTLE, reported no improvement or even worsened outcomes, underscoring the importance of implementation fidelity and staff coverage. PROMPT is also highly cost-effective, especially when refresher courses are repeated, but barriers such as staff release time, leadership buy-in, and resource constraints continue to limit wider adoption. Importantly, high-fidelity simulators are not essential—low-cost props, patient actors, and locally adapted models have proven effective, supporting the programme’s suitability even in low-resource settings. Overall, PROMPT emerges as an effective approach to enhancing the quality of obstetric care and reducing preventable adverse outcomes, provided that its core elements—original training materials, multi-professional participation, regular repetition, and outcome monitoring—are maintained. To maximise its impact, further research is required to overcome methodological limitations, better understand barriers to implementation, and develop strategies to sustain improvements across diverse healthcare systems.

## Data Availability

No datasets were generated or analysed during the current study.
